# ICU admissions and outcomes during the 2014 FIFA World Cup

**DOI:** 10.1186/cc14709

**Published:** 2015-09-28

**Authors:** Décio Diament, Andreia Pardini, Felipe M de T Piza, Guilherme de PP Schettino, Thiago D Corrêa, Flavia R Citadin

**Affiliations:** 1 Adult Intensive Care Unit, Hospital Israelita Albert Einstein, Morumbi, São Paulo, SP, Brazil

## Introduction

There are some data suggesting that stressful events, such as soccer games, could affect the incidence and mortality due to cardiovascular disease. However, data on the impact of large-scale international events on the pattern of ICU admissions and outcomes of critically ill patients are limited.

## Objective

To address the profile and outcomes of the ICU admissions in a private, high-complexity hospital during the 2014 FIFA World Cup held in Brazil, compared with the same period in the previous year.

## Methods

Cross-sectional observational study. All adult patients admitted to a 41-bed medical-surgical ICU of a tertiary care private hospital in São Paulo, Brazil from 12 June to 13 July 2013 (control period) and from 12 June to 13 July 2014 (FIFA World Cup period) were included in this study. Demographic data, SAPS 3 score, clinical and outcome data were retrieved from an electronic ICU quality registry (Epimed Monitor System). Comparisons were performed between the World Cup and the control periods.

## Results

Two hundred and sixty-seven patients were admitted to the ICU during the control period and 251 patients during the World Cup period. The proportion of male patients did not differ between the two periods (58 % vs. 54 %, respectively for control and World Cup periods, *p *= 0.37), as well as the proportion of clinical, elective and emergency surgery admissions (*p *= 0.18). Patients admitted to the ICU during the World Cup period were slightly younger (mean (SD)) than patients admitted during the control period (63 years (±18) vs. 67 years (±18), *p *= 0.031) and had lower SAPS 3 score (45.3 (±15.9) vs. 49.5 (±18.5), *p *= 0.006). The ICU mortality rate was 6.8 % (17/251) for the World Cup period and 6.7 % (18/267) for the control period (adjusted OR, 1.90; 95 % CI, 0.84-4.30; *p *= 0.13). While the median (IQR) length of ICU stay did not differ between the World Cup and control periods (2 (1 to 4) days vs. 2 (1 to 4), respectively, *p *= 0.75), the length of hospital stay was significantly lower during the World Cup period (11 (5 to 28) days vs. 14 (7 to 32) days, *p *= 0.01).

## Conclusion

Although patients admitted to the ICU of a private hospital during the World Cup were slightly younger and less sick compared with patients admitted during the same period in the previous year, the pattern of ICU admissions and the outcomes were not affected. Our results should be compared with those obtained in the other 11 cities selected for the tournament, including private and public hospitals.

